# 520. Polymerized type I collagen as a treatment for COVID-19 in Mexico

**DOI:** 10.1093/ofid/ofad500.589

**Published:** 2023-11-27

**Authors:** Luis Del Carpio-Orantes, Sergio García-Mendez, Jesús Salvador Sánchez-Díaz, Andrés Aguilar-Silva, Manuel Martínez-Rojas, Oscar Rodrigo Jiménez-Flores, Luis Roberto Villalobos-López, América Alejandrina González-Arce, Karem Samantha González-Medel, Rubén Domínguez-Cámara, Alvaro Efrén Munguía-Sereno, Violeta Rosalia Zamora-Vázquez, Quiahuitzin Elizabeth Vázquez-Manzano, Diego Ortíz-Pérez, Semiramis Itzel Hernández-Martínez, Sara Nohemí Hernández-Hernández, Ishar Solís-Sánchez, Victor Alejandro Fonseca-Pouchoulen, Laura Guadalupe Montano-Montiel, Reynaldo Reich-Sierra

**Affiliations:** Grupo de Estudio para el Diagnóstico y Tratamiento de COVID-19 en Veracruz, México, Veracruz, Veracruz-Llave, Mexico; Study Group for the Diagnosis and Treatment of COVID-19 in Veracruz, Mexico, Veracruz, Veracruz-Llave, Mexico; Instituto Mexicano del Seguro Social, Veracruz, Veracruz-Llave, Mexico; Instituto Mexicano del Seguro Social, Veracruz, Veracruz-Llave, Mexico; Instituto Mexicano del Seguro Social, Veracruz, Veracruz-Llave, Mexico; Instituto Mexicano del Seguro Social, Veracruz, Veracruz-Llave, Mexico; Instituto Mexicano del Seguro Social, Veracruz, Veracruz-Llave, Mexico; Instituto Mexicano del Seguro Social, Veracruz, Veracruz-Llave, Mexico; Study Group for the Diagnosis and Treatment of COVID-19, Veracruz, Veracruz-Llave, Mexico; Instituto Mexicano del Seguro Social, Veracruz, Veracruz-Llave, Mexico; Study Group for the Diagnosis and Treatment of COVID-19, Veracruz, Veracruz-Llave, Mexico; Study Group for the Diagnosis and Treatment of COVID-19 in Veracruz, Mexico, Veracruz, Veracruz-Llave, Mexico; Study Group for the Diagnosis and Treatment of COVID-19, Veracruz, Veracruz-Llave, Mexico; Study Group for the Diagnosis and Treatment of COVID-19 in Veracruz, Mexico, Veracruz, Veracruz-Llave, Mexico; Study Group for the Diagnosis and Treatment of COVID-19, Veracruz, Veracruz-Llave, Mexico; Study Group for the Diagnosis and Treatment of COVID-19 in Veracruz, Mexico, Veracruz, Veracruz-Llave, Mexico; Study Group for the Diagnosis and Treatment of COVID-19, Veracruz, Veracruz-Llave, Mexico; Study Group for the Diagnosis and Treatment of COVID-19 in Veracruz, Mexico, Veracruz, Veracruz-Llave, Mexico; Study Group for the Diagnosis and Treatment of COVID-19, Veracruz, Veracruz-Llave, Mexico; Study Group for the Diagnosis and Treatment of COVID-19 in Veracruz, Mexico, Veracruz, Veracruz-Llave, Mexico

## Abstract

**Background:**

In Mexico, Polymerized Type I Collagen (PTIC) has been used as a treatment for patients with moderate to severe COVID-19, who present inflammation, coagulation activation and oxygen requirements, its use being successful both in outpatient and hospital settings, decreasing morbidity and mortality rates. in patients at high risk of complications.

**Methods:**

Descriptive and retrospective study, which analyzes the response to treatment with PTIC throughout the various waves of COVID-19 presented in Mexico, reviewing the inflammation markers and severity indices, as well as the mortality of each group.

**Results:**

250 patients who used PTIC were entered into the study, distributed according to the waves of COVID (2nd wave=35, 3rd wave=65, 4th wave=120 and 5th wave=30), the average age of all the patients was 46 years , being the masculine gender the most prevalent; The main risk factors in this population are: Obesity, Diabetes and Hypertension. Biochemically, all presented lymphocytopenia, mild thrombocytopenia, as well as elevation of inflammatory markers (D-dimer, ferritin, C-reactive protein, neutrophil/lymphocyte index) and high severity scores with data of secondary hypoxemia, highlighting those affected in the 4th wave where the Delta strain predominated. After treatment with PTIC, inflammation and hypoxemia improved significantly, highlighting low mortality rates even during the 4th wave, which was the most aggressive in Mexico and dominated by the Delta-strain. Mortality in each wave was: 2nd wave=2.9%, 3rd wave=3%, 4th wave=6% and 5th wave=0%

**Figure:**
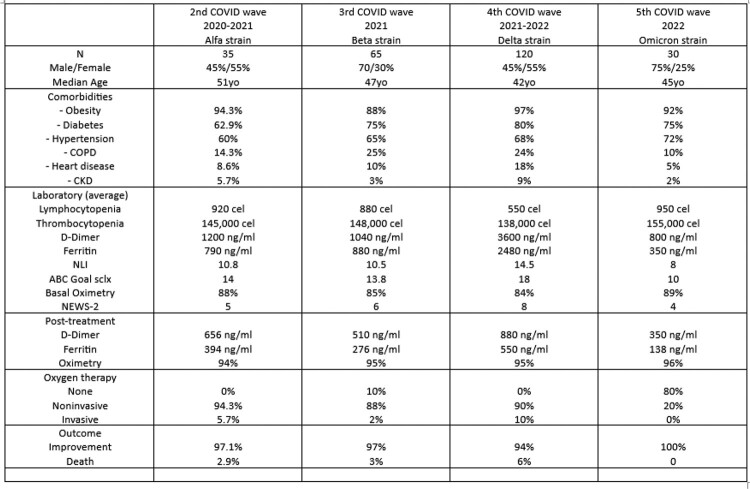

**Conclusion:**

PTIC is a good treatment for moderate to severe COVID-19 in patients with a high risk of complications, significantly reducing mortality rates as well as the risks of inflammation and secondary thrombosis.

**Disclosures:**

**All Authors**: No reported disclosures

